# 11-year experience with Chest Wall resection and reconstruction for primary Chest Wall sarcomas

**DOI:** 10.1186/s13019-020-1064-y

**Published:** 2020-01-28

**Authors:** Ori Wald, Idais Islam, Korach Amit, Rudis Ehud, Erez Eldad, Or Omer, Zik Aviad, Shapira Oz. Moshe, Izhar Uzi

**Affiliations:** 1 Department of Cardiothoracic Surgery, Hadassah Hebrew University Hospital, Jerusalem, Israel; 2 Department of Orthopedics, Hadassah Hebrew University Hospital, Jerusalem, Israel; 3 Department of Oncology, Hadassah Hebrew University Hospital, Jerusalem, Israel

**Keywords:** Primary chest wall sarcoma, Chest wall resection, Chest wall reconstruction

## Abstract

**Background & Objectives:**

Primary chest wall sarcomas are rare and therapeutically challenging tumors. Herein we report the outcomes of a surgery-based multimodality therapy for these pathologies over an 11-year period. In addition, we present a case that illustrates the surgical challenges that extensive chest wall resection may pose.

**Methods:**

Using the Society of Thoracic Surgeons general thoracic surgery database, we have prospectively collected data in our institute on all patients undergoing chest wall resection and reconstruction for primary chest wall sarcomas between June 2008–October 2019.

**Results:**

We performed 28 surgical procedures on 25 patients aged 5 to 91 years (median age 33). Eleven tumors were bone- and cartilage-derived and 14 tumors originated from soft tissue elements. Seven patients (7/25, 28%) received neo-adjuvant therapy and 14 patients (14/25, 56%) received adjuvant therapy. The median number of ribs that were resected was 2.5 (range 0 to 6). In 18/28 (64%) of surgeries, additional skeletal or visceral organs were removed, including: diaphragm [1], scapula [2], sternum [2], lung [2], vertebra [1], clavicle [1] and colon [1]. Chest wall reconstruction was deemed necessary in 16/28 (57%) of cases, polytetrafluoroethylene (PTFE) Gore-Tex patches was used in 13/28 (46%) of cases and biological flaps where used in 4/28 (14%) of cases. R0, R1 and R2 resection margins were achieved in 19/28 (68%), 9/28 (32%) and 0/28 (0%) of cases, respectively. The median follow up time was 33 months (range 2 to 138). During the study period, disease recurred in 8/25 (32%) of patients. Of these, 3 were re-operated on and are free of disease. At date of last follow up, 5/25 (20%) of patients have died due to their disease and in contrast, 20/25 (80%) were alive with no evidence of disease.

**Conclusions:**

Surgery-based multimodality therapy is an effective treatment approach for primary chest wall sarcomas. Resection of additional skeletal or visceral organs and reconstruction with synthetic and/or biological flaps is often required in order to obtain R0 resection margins. Ultimately, long-term survival in this clinical scenario is an achievable goal.

## Introduction

Primary chest wall tumors comprise 1 to 2% of all thoracic neoplasms. They are classified according to their tissue of origin (bone and cartilage vs. soft tissue) and according to their malignant potential (benign vs. malignant). Approximately 60% of primary chest wall tumors are malignant sarcomas, of these, about 55% arise from bone or cartilage and about 45% from soft tissue elements [1]. The clinical presentation of primary chest wall sarcomas is diverse. Indolent tumors slowly grow and are either incidentally diagnosed or present as a painless, palpable mass. In contrast, highly aggressive tumors present as a rapidly growing mass that may invade adjacent thoracic structures such as the lung, mediastinum, vertebra and diaphragm. Consequently, aggressive chest wall sarcomas, often induce pain, shortness of breath, and in some cases, also systemic symptoms [1, 3]. The foundation of treatment for chest wall sarcomas is complete surgical resection to achieve surgical clear margins - R0. In advanced cases, chemotherapy and radiation are utilized either as neo-adjuvant treatments aiming to downstage the tumor and permit resection or as adjuvant treatments aiming to prevent local and systemic recurrence. Metastatic disease is generally considered a contraindication for surgery [2, 4–7]. Given the rare occurrence of primary chest wall sarcomas only a limited number of publications have been published on the long-term outcomes of surgery in this clinical scenario. To expand the available data in this field, we herein report our 11-year experience with chest wall resection and reconstruction for primary chest wall sarcomas.

## Material and methods

### Data collection

Using our departmental prospectively maintained Society of Thoracic Surgeons (STS) database, we identified 25 patients that were operated by a single surgical team (UI, OW) for primary chest wall sarcomas between June 2008 to October 2019. All patients had a confirmed pathological diagnosis. Data on the clinical characteristic and on the outcomes of these patients were prospectively collected and retrospectively reviewed. Complete follow up data was available for 100% of patients. A waiver for patient consent was obtained.

### Patient selection

We performed surgery on patients who’s tumors were considered completely resectable unless their general medical condition did not permit surgery. Metastatic disease was considered a contraindication for surgery. All cases were reviewed by a multidisciplinary team (MDT) prior to surgery. Neo-adjuvant chemotherapy was prescribed according to the MDT decision and adjuvant treatments (chemotherapy and radiation) were considered again by the MDT according to the primary diagnosis, the surgical outcomes and the final pathological report.

### Statistics

Overall survival (OS) was defined as time from date of surgery to death of any cause. Disease free survival (DFS) was defined as time from date of surgery to date of disease recurrence as evident in follow up imaging. Patients were censored on day of last follow up (December 2019). OS is presented using Kaplan-Meier curves.

## Results

### Patient characteristics, tumor histology and adjuvant therapeutics

Between 6 and 2018 to 10–2019, twenty-five patients aged 5 to 91 years (median age 33, male to female ratio 16:9) were diagnosed with primary chest wall sarcomas amenable to surgical resection (Table 1). Eleven tumors (44%) were bone- and cartilage-derived (6 Ewing sarcoma, 4 chondrosarcoma and 1 atypical ossifying fibromyxoid tumor) and 14 (56%) tumors originated from soft tissue elements (5 desmoid, 2 synovial sarcoma, 2 pleomorphic sarcoma, 1 leiomyosarcoma, 1 fibromixoid sarcoma, 1 angiosarcoma, 1 liposarcoma and 1 epithelioid hengioendothelioma). Further, thirteen (52%) of the tumors were low grade (5 desmoid tumors, 3 chondrosarcoma, 1 liposarcoma, 1 hemangioendothelioma, 1 leiomyosarcoma, 1 fibromixoid sarcoma and 1 atypical ossifying fibromyxoid tumor), Three (12%) were intermediate grade (2 synovial sarcomas and 1 chondrosarcoma) and nine (36%) were high grade (6 Ewing sarcoma, 2 pleomorphic sarcoma and 1 angiosarcoma). The median tumor size was 7 cm (range 3 cm to 21 cm). Overall, seven patients (28%) received neo-adjuvant chemotherapy and 14 patients (56%) received adjuvant therapy. Neo-adjuvant chemotherapy was given in six cases of Ewing sarcoma and one case of synovial sarcoma. No pre-operative radiation treatments were administered. With respect to adjuvant treatments, chemotherapy was given in four cases (16%) (2 Ewing sarcoma, 1 pleomorphic sarcoma and 1 angiosarcoma), radiotherapy was given in four cases (16%) (2 desmoid tumors, 1 synovial sarcoma and 1 Ewing sarcoma), chemotherapy and radiotherapy were given in four cases (16%) (3 Ewing sarcoma and 1 fibromexoid sarcoma) and Tamoxifen was given in two cases (8%) for recurrent desmoid tumors. Chemotherapy and radiation protocols were prescribed and administered by the oncology team after discussion in the MDT meeting. Particulate protocols were based on clinical practice guidelines [8].

**Table 1:** This table summarizes the key clinical and histopathological characteristics of the study population. In addition, it summarizes the surgical and therapeutic interventions that each patient had. The table is organized first according to the grade of the tumors and next according to the histological type of the tumor. OS - Overall Survival; DFS - Disease Free Survival; Size - tumor size in centimeters

Age	Sex	OS	DFS	Resection	Primary Dx	Size	Grade	Mode of Repair	Neoadjuvent	Adjuvent
64	Female	10	3	R1	Angiosarcoma	15	High	GoreTex patch + musculocutaneous flap		Chemotherapy + Radiotherapy
16	Male	9	1	R0	Ewing sarcoma	11	High	GoreTex patch + primary repair	Chemotherapy	Chemotherapy + Radiotherapy
10	Female	28	27	R0	Ewing sarcoma	8	High	GoreTex patch + primary repair	Chemotherapy	Chemotherapy + Radiotherapy
31	Male	138	138	R0	Ewing sarcoma	8	High	GoreTex patch + primary repair	Chemotherapy	Chemotherapy + Radiotherapy
12	Female	6	3	R1	Ewing sarcoma	13	High	GoreTex patch + primary repair	Chemotherapy	Chemotherapy + Radiotherapy
5	Male	13	13	R0	Ewing sarcoma	6	High	Primary repair	Chemotherapy	Chemotherapy + Radiotherapy
25	Male	10	10	R0	Ewing sarcoma	20	High	GoreTex patch + primary repair	Chemotherapy	Radiotherapy
91	Female	9	7	R1	Pleomorphic sarcoma	14	High	Primary repair		Chemoterapy
67	Female	80	80	R0	Pleomorphic sarcoma	9	High	Musculocutaneous skin flaps		
66	Male	2	2	R0	Chondrosarcoma	3	Intermediate	Primary repair		
13	Male	27	27	R0	Synovial sarcoma	3	Intermediate	GoreTex patch + primary repair		
18	Male	52	52	R0	Synovial sarcoma	5	Intermediate	Primary repair	Chemotherapy	Radiotherapy
66	Male	3	3	R0	Atypical ossifying fibromyxoid tumor	9	Low	Primary repair		
55	Male	33	33	R0	Chondrosarcoma	6	Low	GoreTex patch + primary repair		
57	Male	119	119	R1	Chondrosarcoma	7	Low	Primary repair		
64	Male	107	107	R0	Chondrosarcoma	8	Low	GoreTex patch + primary repair		
26^a^	Female	123	55	R1	Desmoid	10	Low	Primary repair		Radiotherapy
				R0	Desmoid	6	Low	Primary repair		
25	Male	40	40	R0	Desmoid	3	Low	GoreTex patch + primary repair		
64	Female	48	41	R1	Desmoid	5	Low	GoreTex patch + primary repair		Tamoxifen
54 ^a^	Male	119	24	R1	Desmoid	5	Low	Primary repair		Radiotherapy
				R1	Desmoid	6	Low	Cutaneous skin flaps		Tamoxifen
20 ^a^	Female	135	20	R0	Desmoid	7	Low	Primary repair		
				R0	Desmoid	4	Low	Primary repair		
22	Female	105	105	R0	Epithelioid hemangioendothelioma	7	Low	Primary repair		
35	Male	33	33	R0	Fibromixoid sarcoma	13	Low	GoreTex patch + primary repair		Chemotherapy + Radiotherapy
69	Male	77	77	R0	Leiomyosarcoma	6	Low	Musculocutaneous skin flaps		
71	Male	9	9	R1	Liposarcoma	21	Low	Primary repair		

^a^ These patients had a second operation for disease recurrence

### Operative procedure

We performed a total of 28 surgical procedures on 25 patients. Antibiotics to cover skin microbiota were given for 24 h in the preoperative period regardless of the extent of resection and regardless of the intra-operative use of synthetic patches. The median number of ribs that were resected was 2.5 (range 0 to 6). Further, in 18/28 (64%) of surgeries additional skeletal or visceral organs were removed, including: diaphragm (8), scapula (4), sternum (4), lung (4), vertebra (1), clavicle (1) and colon (1). In addition, in one case, complete dissection of the brachial plexus was performed to release it from an invading synovial sarcoma. Chest wall reconstruction was deemed necessary in 16/28 (57%) of cases. In 13/28 (46%) of cases PTFE Gore-Tex patches were used to reconstruct the skeletal gap in the chest wall and in addition, in 4/28 of cases (14%) rotational flaps were used to cover soft tissue gaps (including 2 latissimus dorsi musculocutaneous flaps, 1 rectus abdominis musculocutaneous flap and 1 rotated skin flap). In all other cases, the wound was primarily closed by skin and subcutaneous tissue flaps that were raised along the incision edge. We routinely inspected the skin incision edge and the biological flaps to confirm their viability (we performed physical examination rather than ultrasound doppler), and when we identified flap engorgement or venous bleeding from the tissue edge we used to irrigate and insufflate the tissue with heparin to permit further oozing and release of tension from the tissue. With respects to gaps in the diaphragm, we were able to primarily repair these gaps by circumferentially suturing of the free diaphragmatic edge to the inferior costal margin. Overall, R0, R1 and R2 resection margins were achieved in 19/28 (68%), 9/28 (32%) and 0/28 (0%) of cases respectively.

### Short-term outcomes

The post operative course was generally uneventful and all patients recovered well from surgery. Five (18%) post-operative complication were recorded within the first 30 postoperative days including, two superficial wound infections, one case of cutaneous flap dehiscence requiring revision, one case of drainage of wound hematoma and one case of deep vein thrombosis. Resolution of these complications was complete in all patients. There was no operative or postoperative mortality.

### Long-term outcome

**Fig. 1:**
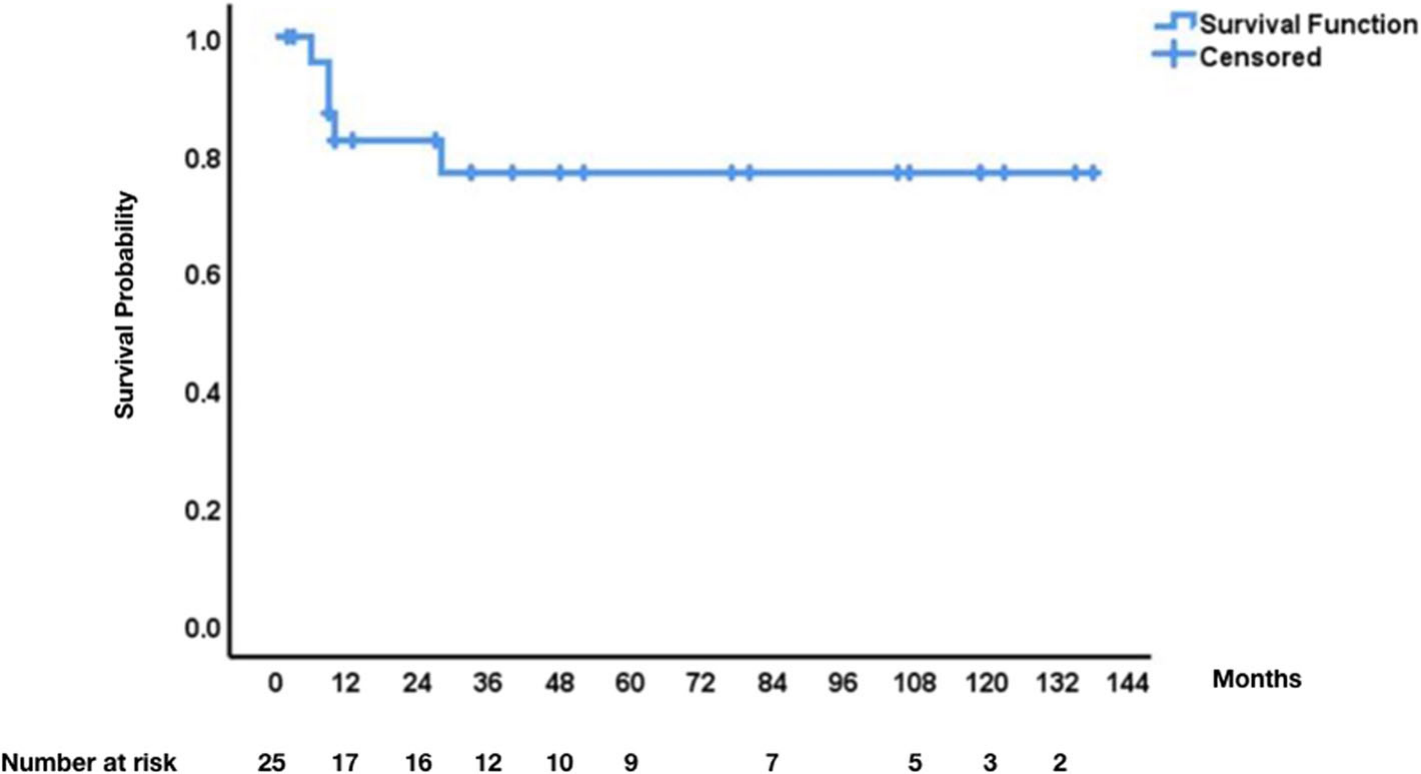
Overall survival of 25 patients with primary chest wall sarcomas

We had a 100% follow up rate on our study population. The median follow up time was 33 months (range 2 to 138). During the study period the disease recurred in 8/25 (32%) of patients. In particular, the disease recurred in three patients with desmoid tumors, three patients with Ewing sarcoma, one patient with angiosarcoma, and one patient with pleomorphic sarcoma. The median time for disease recurrence for desmoid tumors was 24 month (range 20 to 55 months) whereas the median time for disease recurrence in the other sarcomas was 3 months (range 1 to 27 months). Remarkably, the three patients with recurrent desmoid tumor were re-operated on and at date of last follow up, were free of disease. Furtther, at date of last follow up, 5/25 (20%) of patients have died due to their disease (3 Ewing sarcoma 1 angiosarcoma, and 1 pleomorphic sarcoma) and in contrast, 20/25 (80%) were alive with no evidence of disease. Thus, the overall three- and five-year survival rates were 80% (Fig. 1).

## Brief case presentation - extensive chest wall resection for angiosarcoma

**Fig. 2:**
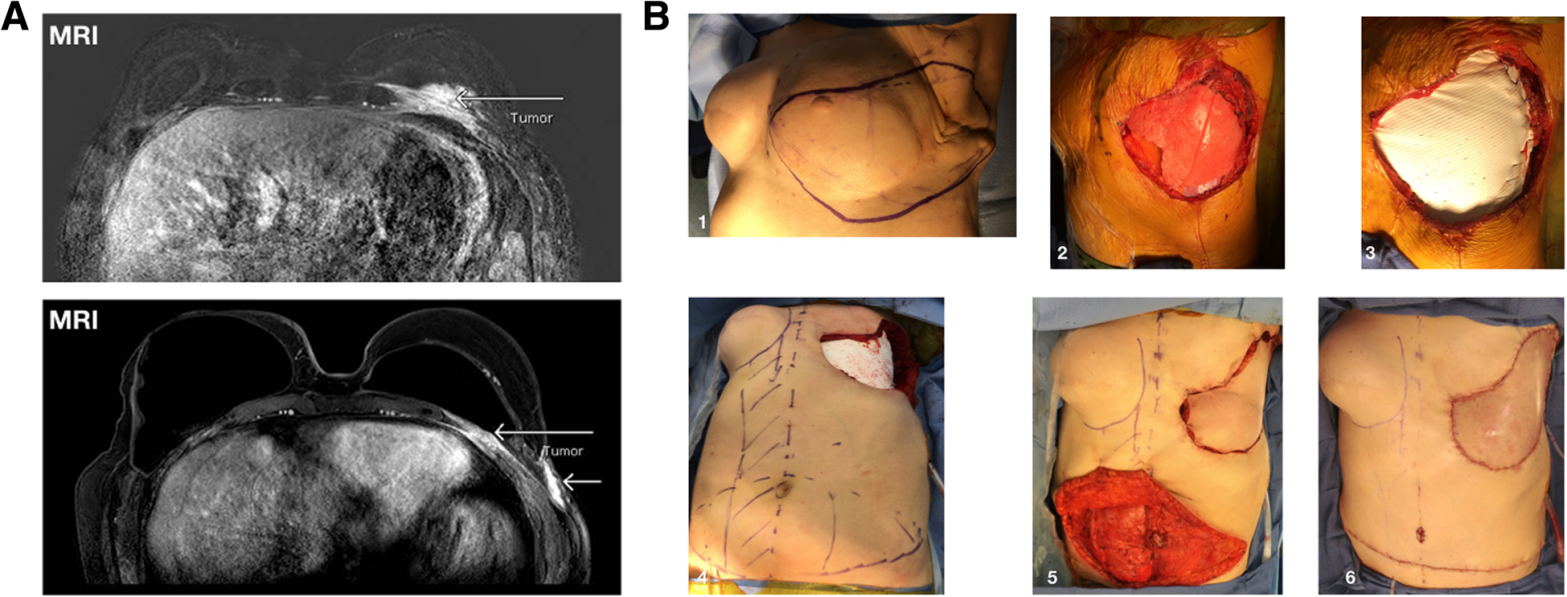
Extensive chest wall resection for angiosarcoma. Shown in A are two MRI sections of a 64-year-old woman with a diagnosis of a high-grade angiosarcoma of the chest wall. Shown in B are: the planed resection margins - B1, the actual gap in the chest wall with exposure of the entire left lung - B2, the reconstruction of the chest wall gap with a PTFE Gore-Tex patch- B3, the planed right rectus abdominis musculocutanous rotational flap - B4, the harvesting, transfer and anchorage of the flap at the surgical site - B5, and the closure of the abdominal site - B6

To illustrate the complexity and challenges involved in the surgical care of patient with chest wall sarcomas we present the case of a 64-year-old woman with a diagnosis of a high-grade angiosarcoma of the chest. In brief, this patient history is notable for a left breast cancer that was diagnosed in 2000. Initially, she underwent a lumpectomy with sentinel lymph node sampling that was negative. However, since the characteristics of her tumor were aggressive she thereafter received adjuvant chemotherapy, hormonal therapy (Tamoxifen) and radiation to the surgical site. Ten years later, she was diagnosed with an intermediate to high-grade angiosarcoma at the surgical site. She then had a formal mastectomy with wide local resection and on pathology the resection margins were clear. Consequently, no further treatments were given. A year later in 2011, she had reconstruction of her left breast using a latissimus dorsi musculocutaneous flap. Unfortunately, in 2017 a large mass involving the deep layers of her left chest wall was seen in follow up MRI - Fig. 2a. Biopsy revealed a high-grade angiosarcoma with MIB proliferation index > 50%. The case was discussed in the MDT meeting where the oncologist and radiotherapists were skeptical about the option of shrinking her tumor with neo-adjuvant chemo-radio-therapy. We therefore offered an upfront salvage resection attempt.

To achieve complete resection we planed our resection margins to extend laterally towards the posterior axillary line and medially towards the center of the sternal bone, we planed to reconstruct the skeletal gap with a PTFE Gore-Tex patch - Fig. 2b (the resection margins - B1, the actual gap in the chest wall with exposure of the entire left lung - B2, and the reconstruction phase with a PTFE Gore-Tex patch - B3 are shown). Further, given that during surgery we anticipated to resect the latissimus dorsi muscle flap and also to sacrifice the left internal mammy artery, we planed to reconstruct the chest wall with a rotated right rectus abdominis musculocutanous flap - Fig. 2b (the planed harvesting of the rectus abdominis musculocutaneous flap - B4, its transfer and anchorage at the primary surgical site - B5, and the closure of the abdominal site - B6 are shown). The surgical course was uneventful and our intra-operative assessment was that we performed the widest possible resection. We estimated that further extension of the resection may endanger the patient with complex wound healing complications. Overall the patient recovered very well from surgery with no complications. On final pathology, the tumor size was 14.5 cm and the resection margins had microscopic islets of tumor at the lateral resection border - R1. Unfortunately the disease macroscopically recurred early thereafter (3 months) and the patients’ overall survival from date of surgery was 10 months.

## Discussion

The preliminary workup of a suspicious chest wall mass includes: 1) a computed tomography (CT) scan and or a magnetic resonance imaging (MRI) of the chest to characterize the lesion and delineate its extent within the thoracic cavity; 2) a core needle biopsy to obtain a diagnosis and ascertain the tumor grade; and 3) a total body CT scan or a positive emotion tomography - CT (PET-CT) scan to search for metastatic spread [1, 3, 9]. Once this evaluation is complete considerations regarding the resectability of the tumor and the need for neo-adjuvant therapy are made. Important factors influencing the design of the treatment plan include tumor histology, grade and size, the potential to achieve clear surgical margins and the general health status of the patient [10–14]. Overall, we performed 28 surgeries on 25 patient. Seven young (age 5 to 31) and generally healthy patients received neo-adjuvant chemotherapy for high and intermediate grade tumors. They all tolerated the treatment well and in 6/7 (85%) of their surgeries R0 margins were achieved. All of these patients also received adjuvant chemotherapy and or radiotherapy. In eighteen patients neo-adjuvant chemotherapy was not administered, either because they had low grade tumors (13 cases) or because their general health status did not permit (2 high grade cases) or because their tumors were considered small enough for upfront complete resection (1 high grade and 2 intermediate grade cases). Among the 18 patients who did not receive neo-adjuvant treatment, R0 margins were achieved in 13/21 (62%) of surgeries. Adjuvant chemotherapy or radiotherapy was given to five patients and two additional patients received Tamoxifen for desmoid tumors. The overall R0 resection rate in our series was 19/28 (68%). When retrospectively evaluating which preoperative parameters might have contributed to R1 resection margins, we identified the following: 1) tumors larger than 10 cm (R1 margins in 5/8 of such tumors); 2) a diagnosis of a desmoid tumor (R1 margins in 4/8 of surgeries for this pathology); 3) Tumor extension to vital anatomic structure such as abdominal aorta and vertebral body; and 4) Intra-operative identification of tumor extension towards a resection margin that, if further extended, may create an unbridgeable gap in the chest wall (see case presentation).

Keeping in mind that achieving clear resection margins remains the key goal of surgery for chest wall sarcomas, we argue that it is important in the preoperative planning to thoroughly consider the desired extent of resection, and also to prepare for the appropriate reconstructive steps [2, 4–7, 10–14]. Overall, in the current series, we have applied an aggressive surgical resection approach. This is evident from the resection of additional skeletal or visceral organs in 64% of cases and from the necessity to reconstruct the chest wall in 57% of cases. And yet, in some instances, the extent of the disease beyond the borders delineated in preoperative imaging was only revealed at time of surgery. In such scenarios, the decision of whether to extend the resection or not is made by the surgical team based on histological examination of snap frozen tissue samples obtained from the resection margins and on the surgeons’ assessment of the consequences of extending the resection (i.e endangering vital organs, chances for surgical site healing complications and functional and cosmetic disability associated with extending the resection) [9]. The decision is often not easy to make and there is no substitute for the experience and expertise of the operating team.

The current report has several limitations, but also certain strengths. In particular, the overall small number of cases that we report on, makes any generalization of our observation difficult. In addition, the histological spectrum of pathologies that we report on is wide and includes both aggressive and indolent tumors. Furthermore, the follow up time on some of the patients is rather short and their age range is wide. Nevertheless, we argue that given the rarity of primary chest wall sarcomas, it is important to report even on a small and diverse number of cases, making the data available for future meta-analyses. Moreover, we consider our data strong with respect to its prospective method of data collection and the 100% follow up that we have on our study population. Finally, given that a single surgical team performed all surgeries the unity in surgical skills and judgment is assured throughout the study.

## Conclusion

In conclusion, a multidisciplinary team of physicians is involved in the evaluation and management of primary chest wall sarcomas. Surgery-based multimodality therapy to achieve and maintain clear resection margins is the foundation of treatment for these pathologies. Long-term survival can be achieved in a significant portion of patients.

## Data Availability

All the data for this study is available and provided in the Table 1.

## Abbreviations

CT: Computed tomography
DFS: Disease free survival
MDT: Multidisciplinary team
MRI: Magnetic resonance imaging
OS: Overall survival
PET-CT: Positive emotion tomography – CT
PTFE: Polytetrafluoroethylene
STS: Society of thoracic surgeons
